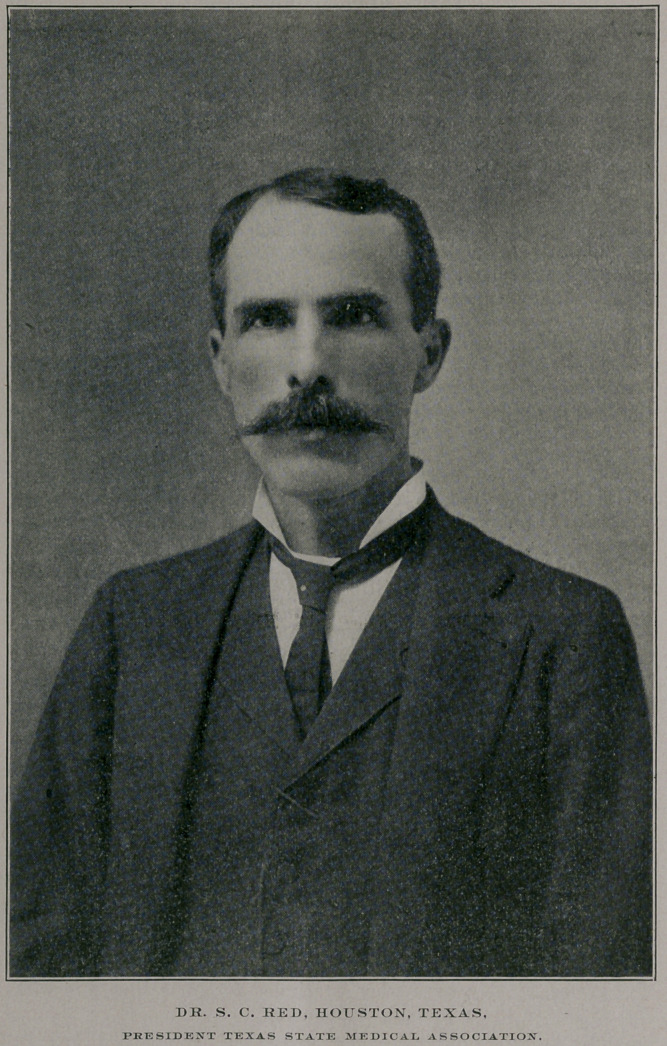# The Public Health

**Published:** 1902-07

**Authors:** 


### TEXAS MEDICAL JOURNAL,

AUSTIN, TEXAS.

A MONTHLY JOURNAL OF MEDICINE AND SURGERY.

EDITED AND PUBLISHED BY

F. E. DANIEL, M. D.

ASSOCIATE EDITOR:

WITTEN BOOTH RUSS, M. D.,

San Antonio, Texas.

Published Monthly at Austin, Texas. Subscription price $1.00 a year in advance.

Eastern Representative: John Guy Monihan, St. Paul Building, 220 Broadway, New York City.

Official organ of the the West Texas Medical Association, the Houston District Medical Association, the Austin District Medical Society, the Brazos Valley Medical Association, the Galveston County Medical Society, and several others.

### THE PUBLIC HEALTH.

**A Plea for Preventive Measures Against Consumption and Other Deadly Diseases in Texas.**

The committee on a State Board of Health, appointed by the Texas State Medical Association, at the Dallas meeting, May 6-9, 1902, under instruction from that body, have issued 7,000 pamphlets of the papers read by Drs. Daniel and Carter (see TEXAS MEDICAL JOURNAL, June, 1902), and an abstract of the speech delivered by Governor Sayers to the Convention, in which he strongly advocated a State Board of Health in place of the present Quarantine Department, as being necessary to deal effectively with the questions discussed in the papers. To these was added a brief note of warning to the people, pointing out the danger of consumption being communicated from the sick to the well, with suggestions of methods of avoiding it. I append this note, as also the introductory, as it appears in the pamphlet. The appropriation of $200 was entirely inadequate to the publication of more than one pamphlet in sufficient numbers to be of any avail, seventy dollars of the amount being required for postage alone on 7,000 copies. It was intended to incorporate in the publication more elaborate information for the people, but to have made the pamphlet larger would have doubled the amount of postage. These pamphlets have been mailed to every newspaper in Texas, every judge, every member of the Bar Association, every representative and senator, as well as all of the defeated candidates for the Legislature, every county commissioner and county physician, as well as to every member of the State Medical Association, and to the secretaries of State Boards of Health in other States. The pamphlet is dedicated to the press and the people in the following words, printed on the first page in red ink:

*"To the Press and the People of Texas:*

"The Press, because it is the great conservator of the Public Weal, and the People, because they are the only legitimate source of power to correct abuses or redress wrongs, this paper is respectfully dedicated.

"THE COMMITTEE."

The following is the Introductory:

*To the Press and the People of Texas:*

We earnestly ask your attention to the plea herein made for action by the State to protect the health and save the lives of the people. We need and must have laws that take cognizance of diseases that originate in our midst and which destroy annually more lives than all the yellow fever, cholera, plague, and smallpox combined; and yet those diseases are the only ones guarded against in Texas. Consumption, for instance (also called *"tuberculosis," "phthisis pulmonalis,"* etc.), *destroys every year fifty per cent. more lives than are lost by yellow fever in a century!* (see Dr. Carter's paper) and yet consumption is preventable. It is impossible to estimate the money value of the 150,000 lives needlessly lost in the United States every year by the great white plague. It would, at the valuation placed upon an able-bodied immigrant, amount to $150,000,000. The whole world is aroused to this great danger, and efforts are being made in all enlightened countries—except Texas—to arrest its progress. In the march of sanitary progress the great State of Texas must be no laggard. The time has come when governors and legislators can no longer shut their eyes to the fact that measures of prevention *must be instituted to protect the people* (who are in ignorance of the danger of such diseases) from *consumption,* typhoid fever, diphtheria, pneumonia, and other deadly diseases; nor shut their ears to the voice of medical science, which proclaims from the housetops, "Those diseases are *communicable,* and can and should be prevented." The expense should be no consideration, and the State should understand at once and for all, that she can not get something for nothin, and that she must pay medical men for their services in the cause of Preventive Medicine (State Medicine, Public Hygiene) as all others are paid. Texas must have a State Board of Health. It is the pressing need of the day. A bill creating such board, framed after the laws now in successful operation in the banner States of Michigan, Massachusetts, and Illinois will be introduced in the next Texas Legislature, and we call upon every intelligent person who reads this pamphlet to use his influence to secure its passage. The people must be enlightened upon the subject of the dangers of communicating consumption from the sick to the well, of acquiring typhoid fever from drinking water from contaminated streams and shallow wells of seep water. The measures of prevention can only be instituted by an intelligent board of health, backed by the authority of the law. First and foremost, the board must institute and put into operation a registration of the vital statistics of the State. This is fundamental, and without it no reform in sanitation is possible. Read this pamphlet; get a friend to read it; get your town paper to publish it, or an abstract of it. Work to help the medical profession in the great cause of reform in our health laws, and for the protection of the public and your own families against the great dangers herein pointed out.

F. E. DANIEL, M. D.,
Editor *Texas Medical Journal,* Austin, Texas, Chairman.
FRANK PASCHAL, M. D.,
City Health Officer, San Antonio, Texas.
BACON SAUNDERS, M. D.,
Dean, Fort Worth Medical College, Fort Worth, Texas.
P. C. COLEMAN, M. D.,
Colorado City, Texas.
W. R. BLAYLOCK, M. D.,
McGregor, Texas.
Texas State Medical Association's Committee on
State Board of Health.

The pamphlet closes with:

### A WARNING TO THE PUBLIC.

"Consumption (tuberculosis) is an infectious disease; so declared by the Tuberculosis Congresses everywhere being held. It is not as "catching" as measles, but it is rapidly and fatally communicated from a coughing consumptive to a well person.

The chief danger is in the dried sputum (spit; expectoration). The deadly germ (*tubercle bacillus*) gets into the dust of the room or car and is thus inhaled into the lungs, and finding the proper conditions, germinates like seed put into good ground. All expectorated matter should be destroyed. Spittoons should be used in which is kept a solution of bichloride of mercury (one part to one thousand parts of water).

A consumptive should not occupy the same room with any other person. The disease may be disseminated by kissing.

The dust in a room or car infected with consumption should not be swept or brushed with a duster, but should be wiped up with a cloth dampened in a one to one thousand solution of bichloride of mercury, and the rag should be burned. Sleeping-car porters often acquire consumption by inhaling the dust.

Sunlight and air *will not disinfect a room or car,* though both are necessary to health, and are advisable. Such rooms or cars, after being occupied by a consumptive, should be fumigated by sulphur or formaldehyde, under directions of an intelligent physician. The State must, and will, provide hospitals for the indigent consumptives who flock to Texas, and thus prevent their scattering the disease broadcast.

Water from suspected sources—principally seep water wells—should be boiled and filtered, to prevent typhoid fever, and all the dejecta of fever patients should be burned or buried."

Before the assembling of the Legislature a bill for a State Board of Health will be carefully drawn up by the committee, under the guidance and advice of the Representatives of Travis county, Hon. A W. Terrell and Hon. J. L. Peeler, both eminent and distinguished lawyers; and it will be introduced in the House by Judge Terrell, who will champion it. When the Legislature assembles an additional copy of the pamphlet will be laid upon the desk of every member, "lest they forget." The committee feel confident of the co-operation and active support of our to-be-Governor, Hon. S. W. T. Lanham, present Congressman, who, it is believed, will especially recommend in his message the creation of a Board of Health.

---

**Abortion Advertisements.**

When we get a Board of Health, which will be when the Legislalature meets in January, one of the things to be attended to, in addition to those enumerated in the pamphlet, will be an effort to suppress the publication in newspapers of abortion advertisements. The subject comes legitimately within the police powers of the State, and the control of the State Board of Health, not as a question of morals, but of public health and social economics. I reproduce from the *Journal of the American Medical Association* the "Kyger

Resolutions," passed by the Kansas City Academy of Medicine,, relative to the abolition from newspapers of "personal advertisements." These resolutions were, by the American Medical Association at the Saratoga meeting in June, referred to the business committee, and will be acted upon at the New Orleans meeting next May. They fully explain the evil, and show the necessity of correction. The to-be-State Board of Health will certainly strenuously co-operate in the efforts to correct it. No more important question could occupy its attention and the attention of the law-making powers. It should be made a penal offense to publish such advertisements, and the papers should be excluded from the mails.

*The Kyger Resolutions*: Personal Medical Advertisements. (From *Journal American Medical Association,* June 21, p. 1653.):

"WHEREAS, It can be and has been shown, by ample statistics, that the American race is rapidly decreasing in its birth rate, thereby threatening ultimate and complete decadence of the race, and

"WHEREAS, Such decadence has become so apparent that it should claim the serious attention of those of influence and power to in any degree lessen this evil, and

"WHEREAS, Without a special effort to investigate, it must have been observed by the most indifferent with what flagrant violation of all sense of delicacy the public press gives place to advertisements of nostrums and means intended to prevent or cut short pregnancy; these advertisements appearing in a column of the paper set apart for such purpose under the name of "Personal Medical Advertisements," and referred to as "Guarantees," "Sure Relief," "Sure Prevention," etc., occupying in some Sunday editions of reputable papers as much as two columns, destined to fall into the hands of all classes, and

"WHEREAS, We recognize the press as a most potent factor in the education of the masses; be it

"*Resolved,* By the Academy of Medicine of Kansas City, Mo., that we respectfully recommend that a censorship over the public press should be exercised to the end of correcting such practice of publishing advertisements as those referred to in our whereases. Be it further

"*Resolved,* That it should be deemed of sufficient moment for the attention of the Postoffice Department of the United States of America restricting or prohibiting the distribution of such papers, periodicals or magazines through the United States mail if they continue to so prostitute their columns with such matter. And be it further

"*Resolved,* That a copy of these resolutions be sent every State

Medical Association in the United States urging their co-operation in this movement by the adoption of these resolutions.

"*Resolved,* That we request the Secretary of every State Medical Association adopting these resolutions to forward two copies, one to the American Medical Association and the other to the Postmaster-General petitioning for relief from this destructive influence.

"JOHN W. KYGER, M. D.,

"H. C. CROWELL, M. D.,

"B. H. ZWART, M. D.,

"Committee."

---

**A Father in Medicine.**

Dr. W. A. Morris, of Austin, died in this city, May 12, 1902, aged ninety years. He practiced medicine more than sixty years, but for the last eight or ten years was retired from active practice. He was one of the organizers of the Austin District Medical Association, and was its second president. Dr. Morris was a native of Henry county, Va. He was a man of exceptionally pure character, and was greatly beloved by all who knew him. He was the preceptor and instructor of many young—now middle-aged—men who have done credit to him and to themselves by their professional career,—amongst them his son, Prof. Seth M. Morris, professor of chemistry in the medical department, University of Texas. The Austin District Medical Society passed resolutions upon his death, paying a high tribute to his character as a physician and a Christian citizen, and, attending his funeral in a body, bore to the grave and placed thereon a beautiful floral offering, a cross and a crown, typifying his labors and his reward.

---

**The State Association's Problem of Problems.**

In our June number there appeared two editorials (one by the senior editor and the other by myself) on the unhappy outcome of the efforts recently made at Dallas to reorganize the State Medical Association. From the text of these articles it is apparent that the senior editor and I differ somewhat as to the manner in which certain matters bearing on this important subject were settled. The points upon which we fail to agree, however, it will be observed, are unimportant, and, besides, are matters of record that can be settled by referenec to a copy of the "Transactions." In the main, I am glad to say, we are entirely agreed in every essential. We both appreciate how vitally important it is that the profession of our State be united in a compact organization and federated through the State association with all of the other State and territorial societies to form the American Medical Association, and I am sure we would both fight to the last ditch a proposition to adopt any radical plan of reorganization that might result in a division of the State into sections, or that might seriously injure the several district societies now in existence.

To review again the question at issue, which is to be settled at San Antonio next April, it will be remembered that the committee of five on constitution and by-laws appointed by President Taylor Hudson some six months ago presented before the convention at Dallas two drafts of a proposed constitution and by-laws, which came to be known respectively as the "majority" and the "minority" reports. The essential features of the *majority report* were: (a) affiliated county and district societies are entitled to representation in the legislative branch of the State society, the number of representatives being determined by their total membership; (b) the members of affiliated societies are to be eligible to membership in the State society, but it is optional with them as to whether they join or not; and (c) the delegate body of the State association may redistrict the State with the consent of the interested local societies. The *minority report* requires that (a) the State be divided into fifteen districts, the distribution of the medical population being taken as the basis of division; and (b) the membership of the county societies of each district shall constitute the district society, and the membership of the several district societies taken together shall in turn constitute the State society.

Upon their presentation, both of these were referred without discussion to a second committee, consisting of about thirty-five members, selected to represent all parts of the State.

The supporters of the majority draft claimed that (1) if the State were arbitrarily redistricted many of the large so-called district societies now in existence would withdraw from the State association rather than yield a part of their territory; and (2) that if membership in the county societies were made to constitute membership in the district society and through it in the State association (a plan of organization similar to that of churches, fraternal orders and of our State government itself) the attendant expense would be so great that many would not be able to keep up their membership.

The advocates of the *minority report* took the ground (1) that to carry out any plan of reorganization it would be absolutely necessary to divide the State into districts, the number of such and their limits of course being immaterial. It would be possible, it was claimed, to redistrict the State without interfering materially with any of the existing societies; (2) that the present plan in our State of allowing every one who happens to belong to one of the affiliated county or district societies to have representation in the State association, whether he was elected to become one of its members and supporters or not (which plan would be continued unchanged if the *majority report* were adopted), is but little less than absurd; (3) that the *majority report* did not conform in any essential particular with the recommendations of the American Medical Association bearing upon this subject.

The committee remained in session or ten or twelve hours, and, section by section, worked out a new draft by a parallel reading of the two originals. Though the advocates of *majority report,* who were in absolute control of the situation, manifested no disposition to abuse their power, they yielded only non-essential points, with one important exception. This exception consists in the incorporation into the new draft of a provision allowing the State association absolute authority to redistrict the State, either with or without the consent of the interested societies. In this particular the original *minority* and the new compromise drafts are in accord. The original majority report alone allows the district societies to decide whether they will submit to a redistricting of the State.

I am under the impression that State Secretary West has been instructed to distribute copies of the two original reports as they were submitted to the convention to begin with. However, I may be mistaken. Instead, it may be that he is to distribute the original minority report, together with the new compromise report. This is again a matter of record, and besides, is of no great consequence. It is already practically assured that no one of the three drafts will be finally adopted without further modification.

This reorganization question is of the utmost importance. It should be talked over earnestly and often until its final settlement next April at San Antonio. There can be no doubt but that the advocates of both of the original drafts have identically the same end in view, viz., the settlement of the question in a manner that will work the greatest good to the profession of the State as a whole. No other motive has ever been or can be ascribed to any one of the leaders on either side. The arguments of both sides should, therefore, be considered in a spirit of toleration and of compromise. No partisan feeling should enter into the discussion. The man who allows the personality of the advocates of either of the two drafts to bias his judgment is not worthy to cast a ballot.

W. B. R.

**A Much Needed Hospital.**

San Antonio is soon to have a model institution for the care of the sick, designed to combine the good features of the so-called sanitarium with those of the general medical and surgical hospital. Thirty of the leading physicians of that city, together with as many or more prominent business men have organized a $50,000 stock company and have purchased a superb property consisting of almost an entire block, centrally and accessibly located in a good neighborhood, just off one of the main thoroughfares.

The architects, Phelps, Shand & King, have completed drawings for the first building, which is to be a modern three-story brick structure, with an elevator, steam heating, electric lights, and every convenience and appliance found in the best applied sanitaria. In addition, the plans provide for a future extension of some two hundred or more rooms.

On the list of stockholders are the names of such well known men as Geo. W. Brackenridge, G. Bedell Moore, Edwin Chamberlain, Floyd McGown, Hon. Marshall Hicks, Thomas B. Palfrey, and others.

Such an institution as this has been much needed in Southwest Texas for the past ten or fifteen years. In future those invalids who have heretofore had to travel hundreds of miles to enter the private hospitals of the North and East will find equally as good accommodation and as skillful nursing provided at this home institution.

---

**The American Tuberculosis Congress.**

The third annual meeting of the American Congress on Tuberculosis was held in New York City on June 2nd, 3rd, and 4th, ult. Delegates were present from most of the States and Territories and from Canada and from several of the Central and South American republics.

Even before the first session was called to order most of the delegates from a distance had suddenly become both sadder and wiser. It was apparent from the start that most of the prominent members of the New York fraternity would take no part in the meeting and that the cause of all the trouble was the manner in which the congress had been organized. It seems that the provisions of the American Medical Association Code of Ethics had been disregarded. In the make-up of the program for this meeting, for example, there had been included a paper entitled "Koch Reviewed," by one C. C. Carroll, M. D., the owner, operator, and extensive advertiser of a certain secret "consumption cure." [It was unanimously rejected, on motion of Paschal, of Texas.—D.]

A body of less determined men would probably have returned home in disgust, forthwith. We are glad, however, to be able to record that with Drs. William Oldwright, of Canada, and Dr. Frank Paschal, of Texas, in the lead, the delegates of regular physicians two hundred strong, held a meeting at once and effected a complete reorganization. Only physicians were elected to office, men of the highest standing in the profession. [For list of officers see elsewhere.—ED.]

The next meeting will be held in St. Louis in 1904, and will, no doubt, have the enthusiastic support of the best men of the profession everywhere throughout the country.

W. B. R.

[Referring to the above, I desire to add that Judge Clark Bell, the organizer of the congress, and who has earned the thanks and gratitude of the profession and the people for his zeal and labors in the cause, promptly resigned when he realized that in organizing the congress he had not met the views of the profession. Judge Bell declined re-election, which we think is unfortunate, and regret. The mistake he made was in asking Governors to appoint delegates instead of asking State medical societies to do so,—a mistake quite possible with a layman.—D.]

---

THE big North Texas Medical Association held a rousing meeting at Fort Worth June 17, 18, 19 (ult.). Many valuable papers were read and thoroughly discussed. The JOURNAL regrets that the program was not received until too late for publication in the June number. Dr. H. K. Leake, of Dallas, read a valuable paper on "Some Recent Cases of Abdominal Surgery." This paper will appear in the Red Back next month. Dr. Leake, it will be remem- *cathedra* on abdominal operations. The next meeting will be held bered, was a student of the great Lawson Tait, and he speaks *ex* in Dallas in December. Dr. H. L. Moore, of Van Alstyne, is secretary.

---

The JOURNAL is pained to record the sudden death, in Hot Springs, June 23rd, ult., of Dr. J. T. Jelks, of that city. Dr. Jelks was famous throughout the United States as a physician and surgeon, and was a distinguished ex-Confederate surgeon.

## Figures and Tables

**Figure f1:**